# Identifying physiological measures of lifetime welfare status in pigs: exploring the usefulness of haptoglobin, C- reactive protein and hair cortisol sampled at the time of slaughter

**DOI:** 10.1186/s13620-018-0118-0

**Published:** 2018-03-02

**Authors:** G. A. Carroll, L. A. Boyle, A. Hanlon, M. A. Palmer, L. Collins, K. Griffin, D. Armstrong, N. E. O’Connell

**Affiliations:** 1 Institute for Global Food Security, Queens University Belfast, Northern Ireland Technology Centre, Malone Road, Belfast, BT9 5HN UK; 2 Animal & Grassland Research & Innovation Centre, Teagasc Moorepark, Fermoy, Co Cork Republic of Ireland; 3 School of Veterinary Medicine, University College Dublin, Belfield, Dublin 4 Republic of Ireland; 4 Life Sciences, University of Lincoln, Brayford Pool, Lincoln, Lincolnshire LN6 7TS UK; 5 School of Biological Sciences, Queens University Belfast, 97 Lisburn Road, Belfast, BT9 7BL UK; 6 Agri-food and Biosciences Institute Pig Unit, Large Park, Hillsborough, Lisburn, County Down BT26 6DR UK

**Keywords:** C-reactive protein, Hair cortisol, Haptoglobin, Pigs, Slaughter, Welfare

## Abstract

**Background:**

Physiological measures indicative of the welfare status of animals during rearing could form part of an abattoir-based animal health and welfare assessment tool. A total of 66 pigs were used in this study, the aim of which was to assess how serum concentrations of haptoglobin (Hp) and C-reactive protein (CRP) (assessed in 51 pigs), and hair concentrations of cortisol (assessed in 65 pigs), measured at or close to slaughter, reflected welfare-related indicators recorded from the animal during its lifetime. These indicators were recorded at intervals between 7 and 21 weeks of age and included assigning scores for levels of tail and skin lesions, recording the presence or absence of certain health issues, and conducting qualitative behavioural assessments (QBA).

**Results:**

Pigs recorded as having tail lesions during their lifetime had higher hair cortisol levels than those with no tail lesions (tail lesions: 47.87 ± 3.34 pg/mg, no tail lesions: 42.20 ± 3.29 pg/mg, *P* = 0.023), and pigs recorded as having moderate or severe tail lesions had higher Hp levels than those with no or mild tail lesions (moderate/severe: 1.711 mg/ml ± 0.74, none/mild: 0.731 mg/ml ±0.10, *P* = 0.010). Pigs recorded as being lame during their lifetime tended to have higher hair cortisol levels than non-lame pigs (lame: 52.72 pg/mg ± 3.83, not lame: 43.07 pg/mg ± 2.69, *P* = 0.062). QBA scores were not associated with any of the physiological measures (*P* > 0.05). Receiver Operator Curve (ROC) analysis was also carried out to get a better understanding of the usefulness of the physiological measures in discriminating animals that had had welfare-related issues recorded during their lifetime from those that had not. Hair cortisol was determined as having ‘moderate’ accuracy in discriminating pigs that were tail bitten on-farm from unbitten pigs (AUC: 0.748) while Hp and CRP were determined to have no meaningful discriminatory ability (AUC < 0.600).

**Conclusion:**

This research should be repeated on a larger scale, but the results suggest that hair cortisol measured at slaughter could provide insight into the welfare status of pigs during their lifetime. Hp may be a useful indicator of tail lesions in pigs. However, further research utilising a greater proportion of severely bitten pigs is required before conclusions can be drawn.

## Background

Meat inspection (MI) is currently under-utilised in its capacity as an animal health and welfare assessment tool [1], and physiological measures of medium to long-term wellbeing could form part of an extended MI procedure [2–4]. Individual physiological measures vary in how they respond to compromised health and welfare [2, 5] and it may therefore be beneficial to assess more than one measure in order to gain a holistic view of herd health and welfare status [6]. The collection of health and welfare-relevant biomarkers should be done non-invasively [5–7], for example, by collecting the samples peri-mortem, at the point of exsanguination, and the chosen measures should not negatively affect slaughter line speed [2].

Evidence suggests that acute phase proteins (APPs) and cortisol may be useful indicators of the lifetime welfare status of pigs. Serum concentrations of APPs are altered during the acute phase response, a non-specific immune reaction [5]. Concentrations of positive APPs increase in response to issues such as inflammation, trauma, infection or stress, remaining elevated for a number of days [7, 8]. Furthermore, the presence of chronic inflammation, such as that induced by tail lesions or arthritis, can result in a longer-term elevation of positive APPs [9, 10]. Haptoglobin (Hp) and C-Reactive Protein (CRP) are two positive inflammatory markers in pigs [7, 10] and it has been suggested that the combined assessment of these APPs may improve sensitivity in discriminating healthy from unhealthy animals [2].

Cortisol is a physiological measure of stress [11], and levels in urine, saliva and blood have been examined to determine the effects of acute stressors such as mixing, transportation or lairage time [5, 12, 13]. However, cortisol levels fluctuate according to factors such as time of day or food intake [14, 15], and stress induced by invasive collection procedures can also affect cortisol levels [16]. Furthermore, if long-term stress is to be assessed, repeated sampling is necessary. This is labour-intensive and logistically difficult to conduct [15], particularly when animals must be habituated to having samples taken [5]. Hair cortisol, on the other hand, is a measure of chronic stress [16] and has several advantages over traditional cortisol assessment methods; samples can be collected non-invasively, are easy to transport and store, and reflect long-term cortisol levels without the need for repeated sampling [17–19]. Therefore, cortisol levels present in pig hair immediately prior to slaughter may be a useful measure of lifetime welfare in pigs.

Recent research highlighted that healthy pigs at slaughter can be discriminated from those with health conditions (e.g. lung lesions and abscessation), and those likely to be partially or fully condemned [4, 20, 21], through examination of APP levels. Furthermore, APP levels measured at slaughter were used to discriminate healthy pigs from those with health issues (e.g. diarrhoea, earache and external injuries) detected in the period immediately prior to transportation to slaughter [2]. However, there is a lack of information on the extent to which these measures reflect long-term health and welfare-related issues present on-farm. Furthermore, the potential of including hair cortisol as a biomarker of health and welfare at slaughter has yet to be explored. The aim of this study was to determine if a relationship existed between physiological measures recorded close to the time of slaughter (serum Hp and CRP, and hair cortisol) and indicators of welfare recorded from pigs during their lifetime.

## Method

Data were collected between June and December 2014 at the Agri-Food and Biosciences Institute in Hillsborough, Northern Ireland, and at a local commercial abattoir. In total, 66 pigs were assessed over two batches, with each batch being reared at 6-week intervals. Hair samples were available for 65 pigs. Due to issues associated with slaughter line speed, some blood sampling opportunities were missed. As a result, a total of 51 samples were available for Hp and CRP analyses, respectively.

### Animals and housing

Study pigs were PIC 337 breed and had 50% of their tail docked on the day of birth. Pigs were weaned at 28 days and transferred to growing accommodation. During the growing phase, approximately half of weaner pigs were housed in ‘enriched’ pens with deep straw bedding and a space allowance of 0.47m^2^. The remaining piglets were housed in ‘barren’ pens, which had no straw and a space allowance of 0.38m^2^ per pig. At approximately 67 days, pigs were transferred to finishing accommodation. During finishing, pigs were regrouped and housed in fully slatted pens at a space allowance of 0.64m^2^ per pig. The pigs were housed in groups of either 10 or 20 animals. All groups were balanced for sex and weight throughout the study period.

### Collection of lifetime health and welfare measures

Each pig was evaluated on two occasions during the weaning period (at 7 and 9 weeks of age) and on two further occasions during the finishing period (at 15 weeks and prior to slaughter at 20 or 21 weeks of age [depending on slaughter date]).

Each health and welfare evaluation was conducted over two consecutive days per assessment week. Each pig was spray marked to allow for individual identification. In order to conduct physical welfare assessments, each animal was slowly circled and assigned a score for tail lesions, skin lesions and health issues (excluding coughing). Any lying or sitting pigs were encouraged to stand and walk. Pigs unable to stand were not disturbed and only immediately visible welfare issues were recorded. When this occurred, any remaining measures were recorded as ‘missing’. On day 2, behavioural assessments (and assessments of coughing) were conducted. Coughing was scored on day 2 while conducting behavioural observations in order to allow sufficient time for its detection. Pens and pigs within pens were assessed in randomised order. Behavioural observations within the weaning unit took place from video recordings, and observations within the finishing unit were made directly. Pigs were allowed a 2-min ‘settling’ period after the observer entered the house before direct observations commenced. Each pig was weighed at 4 and 10 weeks of age and again 1 day prior to slaughter. Each batch of pigs was split into two groups for transportation to slaughter. Therefore, there were four transportation groups in total.

### Tail lesions

**Fig. 1:**
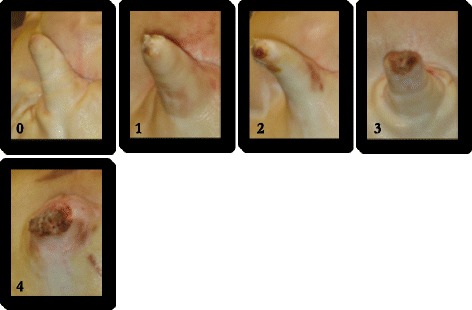
Tail lesion scoring system. (0) no evidence of tail biting (1) mild/healed lesions (2) evidence of chewing or puncture wounds, but no evidence of swelling (3) evidence of chewing or puncture wounds, with swelling and signs of possible infection (4) partial or total loss of tail

Each pig was scored for tail lesions using an adapted version of Kritas and Morrison’s [22] tail scoring system (Fig. 1). For analysis, this tail lesion scoring system was simplified; score 0 was classified as ‘absent’, scores 1 and 2 were combined to form a ‘mild’ tail lesions category, and score 3 and 4 were combined to form a moderate (score 3)/severe (score 4) category.

### Skin lesions

Skin lesions were assessed on 12 regions of the body including the left ear, right ear, snout, left shoulder, right shoulder, front legs, back legs, left flank, right flank, left hindquarter, right hindquarter and back. Each region was scored on a 6 point scale (adapted from Manciocco et al. [23], Conte et al. [24] and Calderón Díaz et al. [25]) which ranged from (0) ‘no injuries’ to (5) ‘many very big, deep and red lesions covering the skin area’. Skin lesion scores assigned on-farm were simplified for analysis and were broken down as ‘absent’ (all regions scoring 0), ‘mild’ (regions scoring 0 to 2 with a maximum of 4 regions scoring 3), ‘moderate’ (regions scoring 0 to 3 with a maximum of 2 regions scoring 4 or 1 region scoring 5) and ‘severe’ (regions scoring 0 to 3 with 3 or more regions scoring 4 or 2 or more regions scoring 5).

### Health issues

Bursitis, hernias, rectal prolapse, aural haematomas and body condition were assessed using the Welfare Quality® [26] protocol. Further information on this protocol can be found at http://www.welfarequalitynetwork.net. Lameness, scouring and coughing were scored using a scoring system adapted from the Welfare Quality® [26] protocol and are described in Table 1. Health condition scores were simplified for analysis; score 0 was classified as ‘absent’ and score 1 or above was classified as ‘present’.

**Table 1:** Health issue scoring systems adapted from Welfare Quality®^a^

Measure	Score	Description
Lameness	0	Normal gait
	1	Difficulty in walking, but still using all legs
	2	Severely lame, minimum weight–bearing on the affected limb
	3	No weight–bearing on the affected limb or not able to walk
Scouring	0	No evidence of scouring
	1	Possibly present by diarrhoea/staining around and below anus
	2	Observed in the act of scouring
Coughing	0	Absent
	1	Present (once)
	2	Persistent (more than once)

^a^Welfare Quality® protocol for pigs [26]

### Behavioural assessments

Behaviour was assessed from outside the pen using Qualitative Behavioural Assessment (QBA) [27]; Each pig was scored on 5 positive behavioural descriptors (‘happy’, ‘content’, ‘positively occupied’, ‘relaxed’, ‘playful’) and 5 negative descriptors (‘frustrated’, ‘irritable’, ‘agitated’, ‘aggressive’, ‘listless’). A scale from 0 to 100 was used where 0 was the lowest possible score and 100 was the highest possible score. For example, an extremely ‘aggressive’ pig would receive a score of 100 for this particular behavioural descriptor.

### Collection of physiological measures

Hair samples were collected between 9 am and 11 am on the day prior to slaughter. On the day of slaughter, pigs were transported 41.3 miles from the farm to the abattoir at 7:00 h with an approximate journey time of 70 min. Pigs were held in the lairage area for between 30 and 60 min and blood samples were collected between 8.30 am and 9.30 am. The methods used for the collection and analysis of samples are described below.

### Hair sample collection procedure

Hair samples were collected while each pig was held in a weighbridge. Using surgical scissors, hair was collected in equal amounts from the left and right sides of the rump area. Hair was cut as close to the root as possible. Each hair sample was placed in a sealable plastic bag and stored at 4 °C. Once all experimental pigs had been sampled, the hair from each animal was transferred to a tin foil pouch and kept at room temperature pending analysis.

### Analysis of hair samples

Samples were washed and treated using the methods described by Davenport et al. [14] for rhesus macaques and Martelli et al. [18] for pigs, with the following adjustments; hair samples were washed in isopropanol a number of times to remove any external contamination. Each sample was then dried and cut into 1–2 mm sections with scissors. Sixty milligram of the samples was then weighed into a 4 ml Tallprep tube (MP Biomedicals, California, USA). Five ceramic balls were added to each tube and the samples were pulverized to a fine powder using a Fastprep 24 instrument (MP Biomedicals, California, USA). Two millilitre methanol was added to the samples and the vial was incubated overnight at room temperature with constant gentle agitation for steroid extraction. Following extraction, methanol was transferred to a fresh vial, evaporated in a scanvan vacuum centrifuge and stored at − 80 °C. Subsequently, EIA analysis was performed according to the method employed by Davenport et al. [14]. Intra-assay and inter-assay coefficients of variation were 7.79% and 0.85%, respectively. The approximate limit of detection for this procedure was 0.007 μg/dL.

### Blood sample collection procedure

Blood samples were taken from each pig by placing a labelled plastic cup at the throat of each animal at the point of exsanguination. The collected blood was then transferred immediately to Z Serum Clot Activator Vacuette tubes (VWR International Ltd., Leicestershire, UK) and stored in a cool box during transfer to the laboratory. Each sample was centrifuged at 3000 rpm for 15 min at 4 °C. One millilitre of serum was obtained and maintained at − 20 °C pending analysis.

### Blood samples analysis

Concentrations of Hp and CRP were determined in duplicate using commercial assay kits (Tridelta Development Ltd., Wicklow, Ireland) in accordance with the manufacturer’s instructions, and were read on a Dynex MRX plate reader. Hp and CRP were expressed in mg/ml and μg/ml, respectively.

### Statistical analysis

#### Outliers

Hp, CRP and cortisol values were examined for outliers using the Outlier Labelling Rule [28]. A number of outliers were detected and the data were log transformed. Subsequent to transformation, there were no outliers and all samples were included in the analysis.

### Pearson’s Chi Square

A number of 2 × 2 Chi Square tests were conducted to determine whether the presence or absence (P/A) of health issues, tail lesions or skin lesions on farm occurred independently of each other. This was done in order to account for the possible effects of co-occurrence of welfare issues, at the individual level, on concentrations of Hp, CRP and hair cortisol.

### Principal component analysis (PCA)

PCA was carried out on all QBA scores. This procedure groups associated variables together into smaller structures and allows the main themes within the data to become interpretable. Based on examination of the scree plot, two principle components (PCs) were extracted. The direct oblimin factor rotation was selected and a Kaiser-Meyer-Olkin (KMO) test value of 0.904 established the appropriateness of PCA analysis. The nature of each PC was interpreted by examination of the pattern matrix. A factor loading cut-off point of 0.3 was applied. Two principal components (PCs) were identified and were entered into fixed effect models.

### Fixed effect models

It is worth noting that, although not presented in the current paper, tail and aggression-related skin lesion scores were recorded from carcasses after scalding and dehairing at the abattoir (e.g. Carroll et al. [29]), and levels of carcass condemnations monitored. Data indicating relationships between these measures and physiological measures recorded close to slaughter were not presented because: (i) there were no partial or whole carcass condemnations, (ii) tail lesions scores measured from the carcass were found to be highly collinear with those recorded during lifetime, and (iii) aggression-related skin lesions on the carcass (‘fresh’ or ‘healed’) were not found to relate to physiological measures at slaughter.

Three linear fixed effects model procedures were carried out to examine the influence of predictor variables in explaining the serum concentrations of Hp and CRP, and hair concentrations of cortisol. Several control and predictor variables, described below, were entered into preliminary models and descriptive statistics were used to explore the data. Due to an absence of some of the more severe welfare scores, a number of predictor variables were condensed into smaller categories. Initially, the group in which animals were transferred to slaughter (labelled ‘transportation group’) was added as a random factor in order to control for the possible effects of transport on levels of Hp and CRP. However, short transport time, as seen in the current study (70 min), would not appear to be associated with increased APP levels due to the time taken for them to become elevated [30]. As expected, the effect of ‘transportation group’ was non-significant and this random factor was removed from the final models.

Variables were entered into each model in stages, with control variables being entered into the initial model. The control variables entered into the preliminary models included included sex, weight at weaning, finishing and slaughter, diet in the growing and finishing periods, batch, weaning enrichment and housing unit. All control variables were non-significant subsequent to backward selection and none were retained. Predictor variables were then entered into the models and included; ‘tail lesion (TL) P/A’ (Present or absent in the lifetime), ‘TL moderate/severe’ (P/A of moderate or severe TL in the lifetime), ‘skin lesion (SL) moderate/severe’ (P/A of moderate or severe SL in the lifetime), ‘P/A of lameness’, ‘P/A of bursitis’, ‘P/A of coughing’, ‘P/A of hernias’, ‘P/A of rectal prolapse’, ‘P/A of scouring’, ‘P/A of aural hematomas’, ‘P/A of poor body condition’, ‘P/A of any health issue’, ‘weaning unit QBA PC1 (principal component 1) score’, ‘weaning unit QBA PC2 score’, ‘finishing unit QBA PC1 score’ and ‘finishing unit QBA PC2 score’.

Collinear variables were entered into models individually and non-significant predictor variables were not retained. Variables ‘Farm TL P/A’ and ‘Farm TL moderate/severe’ were found to be collinear, having a VIF value over 3.0. However, these were entered separately into preliminary models and only one of the two variables was included in any final model by selecting the variable that contributed most to explaining the variation within the model. There was no other retained variable pair with a VIF value over 3.0. No interactions were included within the models. Backward selection was used to eliminate predictor variables until only those with a *P* < 0.05 remained within the model.

The final models included the following predictor variables; ‘Farm tail lesion P/A’, ‘Farm TL moderate/severe’ and ‘P/A of lameness’.

### Receiver operator curve (ROC) analysis

The diagnostic performance of Hp, CRP and hair cortisol in detecting ‘TL P/A’, ‘TL moderate/severe’, ‘SL moderate/severe’, ‘P/A of lameness’, ‘P/A of bursitis’, ‘P/A of coughing’, ‘P/A of hernias’, ‘P/A of rectal prolapse’, ‘P/A of scouring’, ‘P/A of aural hematomas’, ‘P/A of poor body condition’ and ‘P/A of any health issue’ were explored. ROC analysis allows for the identification of cut-off values for discriminating ‘healthy’ animals from those with a health or welfare issue, sometimes referred to as ‘suspects’. Cut-off values that maximise sensitivity and specificity were selected [4]. The Area Under the Curve (AUC) value specifies diagnostic accuracy, with values of ≤0.500 being ‘meaningless’; 0.500–0.700 being ‘less accurate’; 0.700–0.900 being ‘moderately accurate’ and 0.900–1.000 deemed ‘perfect’ [4, 31]. Physiological measures were not examined in combination when assessing ability to predict tail lesions, skin lesions, health issues and QBA scores due to only one of these tests having an AUC value of 0.700 or above.

Alpha level for determination of significance was 0.05. All statistical analyses were carried out using SPSS version 20.

## Results

The descriptive statistics for each physiological measure can be seen in Table 2.

**Table 2:** Descriptive statistics for physiological measures

Measure	Number	Mean	SEM	Min	Max
Hp serum concentration (mg/mL)	51	0.87	0.13	0.05	5.24
CRP serum concentration (μg/mL)	51	313.94	96.7	10.30	4688.90
Hair cortisol (pg/mg)	65	45.73	1.97	11.10	60.80

The most commonly recorded health and welfare issues were tail lesions, skin lesions, coughing, lameness and bursitis. The distribution of each of these welfare issues across the lifetime of the pigs can be seen in Table 3. Twenty pigs had more than one condition. The most common combination of health issues seen in individual pigs were lameness and coughing (5 pigs). Overall, tail lesions were mild, moderate and severe in 69.8%, 22.7% and 7.5% of cases, respectively. Skin lesions were mild, moderate and severe in 93.0%, 6.1% and 0.9% of cases, respectively. Lameness was mild in 82.8% of cases, and moderate in 17.2% of cases. There were no severe cases of lameness detected. Coughing was recorded as being ‘present’ in 71.4% of cases, and ‘persistent’ in 28.6% of cases. All incidence of bursitis were mild.

**Table 3:** Prevalence and severity of the most common welfare indicators across the lifetime observations

Measure	Week 7	Week 9	Week 15	Week 20/21
Prevalence (%)				
Tail lesions				
Mild	4.5%	6.1%	7.6%	16.7%
Moderate	0%	0%	0%	10.6%
Severe	0%	0%	0%	3.0%
Skin lesions				
Absent/mild	100%	100%	92.4%	90.9%
Moderate	0%	0%	1.5%	9.1%
Severe	0%	0%	0%	0%
Lameness				
Mild	1.5%	0%	6.1%	15.2%
Moderate	0%	0%	0%	3.0%
Severe	0%	0%	0%	0%
Cough				
Present	0%	0%	19.7%	4.5%
Persistent	0%	0%	6.1%	1.5%
Bursitis				
Mild	0%	1.5%	7.6%	1.5%
Severe	0%	0%	0%	0%

*N* = 66

### Pearson’s Chi Square

There was no significant relationship between the P/A of any two welfare measures (tail lesions, skin lesions or health issues) at an individual level (*P* > 0.05).

### Qualitative Behavioural analysis

Two principal components of behaviour were identified. Principal components 1 and 2 explained 69.4%, and 11.5% of the variance in QBA scores, respectively. The factors loading onto each component are shown in Table 4. All five positive behaviours loaded strongly onto PC1 and 4 negative behaviours loaded strongly but negatively onto PC1. Therefore, PC1 was labeled as representing ‘Good welfare’. ‘Aggressive’ loaded strongly onto PC2. ‘Listless’ also loaded moderately, but negatively, onto this PC. This PC was labelled as ‘Aggressive’ as this seemed to be the predominant characteristic being represented.

**Table 4:** Descriptor loadings within the two identified principal components (PC)

Descriptor	Principal components	PC1	PC2
Happy		**.966**	.088
Content		**.961**	.065
Positively occupied		**.926**	- .006
Relaxed		**.851**	−.071
Playful		**.914**	.115
Frustrated		**−.805**	.278
Irritable		**−.857**	.252
Agitated		**−.861**	.156
Aggressive		−.156	**.900**
Listless		**−.659**	**−.421**

Factor loadings over 0.3 are highlighted and were used to interpret the structure of each component

### Fixed effects models

#### Tail lesions

Pigs recorded with moderate or severe tail lesions at least once (‘TL moderate/severe’) had higher Hp levels (1.711 mg/ml ± 0.74) than those with no or mild tail lesions (0.731 mg/ml ±0.10, *P* = 0.010). Pigs recorded with any tail lesions (‘TL P/A’) had higher cortisol levels (47.87 pg/mg ±3.34) than those with no tail lesions (42.20 pg/mg ± 3.29, *P* = 0.023). Tail lesions were not a predictor of the levels of serum CRP (*P* > 0.05).

#### Health issues

Pigs that were recorded as lame on at least one observation week tended to have higher cortisol levels (52.72 pg/mg ± 3.83) than those with no recorded lameness (43.07 pg/mg ± 2.69, *P* = 0.062). Lameness was not a predictor of the levels of serum Hp or CRP and no other health issue predictor affected Hp, CRP or hair cortisol levels (*P* > 0.05).

#### Qualitative behavioural assessment

Hp, CRP and hair cortisol concentrations were not associated with any QBA scores (P > 0.05).

### ROC analysis

#### Tail lesions

**Fig. 2:**
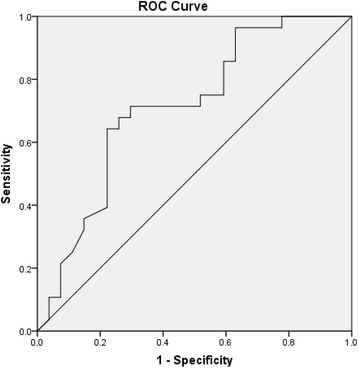
ROC curve of hair cortisol concentrations for distinguishing between pigs that had been tail bitten on-farm to those that had not been tail-bitten on-farm

The ROC analysis for hair cortisol suggested that the most useful cut-off value for discriminating pigs that were tail bitten on-farm from those that were unbitten was 49.9 pg/mg where the sum of sensitivity (73.9%) and specificity (71.4%) was the highest. The area under the curve was 0.748, reflecting ‘moderate’ accuracy in detecting tail bitten pigs (see Fig. 2).

The ROC analyses for Hp suggested that the most useful cut-off value for discriminating pigs that were tail bitten on-farm from those that were unbitten was 0.455 mg/ml, where a sensitivity of 73.9% and specificity of 33.3% was achieved. The ROC analyses for CRP suggested that the most useful cut-off value for discriminating pigs that were tail bitten on-farm from those that were unbitten was 0.90.6 μg/ml where the sum of sensitivity was 73.9% and the sum of specificity was 33.3%. The area under the curve was 0.581 for Hp and 0.565 for CRP respectively, indicting an ability in discriminating tail-bitten from unbitten pigs that is just above chance level.

#### Skin lesions and health issues

Area Under the Curve (AUC) values were < 0.500 for discriminating between the P/A of moderate/severe skin lesions and the P/A of individual health issues for all three physiological measures, indicating that the ability of the physiological measures to detect the presence of skin lesions and health issues on farm was less than that of chance.

## Discussion

### Mixed models analyses

#### Hair cortisol and physical welfare-related measures

The results show that pigs with tail lesions and lameness (trend) had elevated cortisol levels relative to control animals. This finding is supported by previous studies, which examined welfare-related lesions and measures of stress. For example, Munsterhjelm et al. [32] found that victims of tail biting had increased adrenal weights compared to unbitten control pigs, a morphological characteristic associated with chronic stress [33]. Further, Valros et al. [34] found that tail bitten pigs had a lower serum concentration of cortisol after stunning than unbitten pigs, a potential indicator of hypocortisolism which is linked to long-term and repeated stress [34]. Lameness was the only on-farm health issue associated with increased hair cortisol levels, albeit at a level that did not reach statistical significance. Research suggests that lame pigs may be more likely to become victims of tail biting [35] and it is possible that lame pigs also tended to be tail bitten. However, statistical analysis revealed no association in individual pigs between the presence of tail lesions and lameness. Tail lesions and lameness may have been stressful for a number of reasons. For example, avoidance of pen mates by tail bitten pigs may be stressful, particularly when the animal is constantly targeted by an ‘obsessive’ tail biter [36].

It is also possible that the elevated cortisol levels seen in lame and tail-bitten pigs reflected pain, and, in fact, elevated levels of hair cortisol are linked to chronic pain in humans [37]. In pigs, tail lesions are likely to be particularly painful due to the prolonged and repeated nature of tail biting [34]. Similarly, lameness in pigs is associated with physical indicators of pain. For example, injecting lame pigs with an analgesic, meloxicam, resulted in an improved step frequency and an increase in standing posture [38, 39], suggesting that pain experience altered these characteristics.

#### APPs and physical welfare-related measures

According to mixed models analysis, Hp was elevated in pigs with moderate to severe tail lesions, while CRP levels were unaffected by tail lesion status. This finding differs to that of Heinonen et al. [10] who found that tail lesions were associated with elevated levels of both Hp and CRP measured at slaughter. All tail lesions examined by Heinonen et al. [10] were deemed severe, with partial loss of the tail length evident in all cases. However, severe lesions were less common in the current study. Different APPs can also vary in the extent to which they react to the same stimulus (e.g. Saco et al. [40]) and it may be that Hp is a more sensitive indicator of the presence of tail lesions than CRP.

Positive APP levels increase not only in response to inflammation and infection [41], but to psychological stressors such as transportation and rehousing [7]. The fact that only the more serious tail lesions were associated with increased Hp levels may have been due to the degree of inflammation and infection associated with more severe lesions (Fig. 1), or increased pain experienced. However, to date, very little research has been conducted into the effects of tail lesions in pigs on levels of different APPs and further research in this area is needed before final conclusions can be drawn.

In contrast to hair cortisol levels, lameness did not appear to be associated with APP levels measured at slaughter. Indeed, levels of Hp or CRP were not affected by skin lesion score or any other health issue examined.

In many cases, skin lesions, lameness and other health issues were in their milder form and were only seen on one or two observation weeks (Table 3). Consequently, the acute phase response associated with the recorded health or welfare issue may have subsided by the time of slaughter. In contrast, hair cortisol levels can reflect a period of several months [15] and this may explain why an increase in hair cortisol levels tended to be evident in pigs with a history of lameness. This perhaps highlights the importance of assessing a variety of physiological measures at slaughter in reflecting the longer-term welfare status of pigs.

### ROC curve analyses

The ROC curve analyses revealed that hair cortisol had ‘moderate accuracy’ in discriminating tail bitten from non-tail bitten pigs. Apart from this, no physiological measure was deemed able to discriminate between healthy and suspect animals in a meaningful way. For example, while Hp demonstrated a statistically significant association with risk of being tail bitten in multivariate models, it did not discriminate between bitten and unbitten pigs in predictive models. On some occasions, a compromise between sensitivity and specificity is required that will be context-specific [31]. A sensitivity of 73.9% and specificity of 33.3% was achieved for Hp in discriminating bitten from unbitten pigs. High levels of sensitivity were deemed more important in the current study than high levels of specificity. This is because it may be most important to correctly identify pigs that have been bitten (sensitivity) than to correctly identify those without tail lesions (specificity). However, a specificity of 33.3% means that two thirds of ‘healthy’ pigs would be incorrectly identified as having been tail-bitten. This indicating that this measure would not be of practical use in informing farmers of potential tail biting issues within the herd as feedback would be inaccurate in many cases.

Mixed model analysis revealed that Hp was associated with moderate/severe tail lesions. However, in comparison to the above, ROC curve is a relatively crude analysis, simply measuring the usefulness of a test in general [42]. Therefore, more complex levels of lesion severity cannot be considered. This may explain this finding; as can be seen in Table 3, only 3% of pigs had severe tail lesions. Future research using a greater number of more severely tail bitten pigs is needed if the ability of APPs to discriminate between bitten and unbitten pigs in general, i.e., when assessing pigs from various farms and housing systems, is to be confirmed.

### Physiological measures and qualitative Behavioural assessment

Subjective behavioural measures such as QBA can be susceptible to observer bias [43] and the validity of QBA as a measure of animal welfare remains in question [44]. Recently, a number of studies revealed an association between QBA and physiological measures such as heart rate and body temperature [45–47]. However, no associations between QBA and physiological measures were found in the current study. Previous studies utilising QBA have created distinct experimental groups, for example, by comparing the behaviour of pigs that have been administered a saline solution to those that have been administered a behaviour-altering drug [46]. Differences between groups in such cases are likely to be conspicuous. However, in the current study, differences the behaviour of individual pigs were not as distinctive. This may have been due to a lack of more severe forms of the various health and welfare issues that were observed.

## Conclusions

Hair cortisol appears to be a useful physiological measure of lifetime welfare status in pigs, reflecting tail lesions, and potentially, lameness on-farm. Hp may be a useful indicator of tail lesions in pigs. However, further research utilising a greater proportion of severely bitten pigs is required before conclusions can be drawn. It is possible that the Acute Phase Response may subside before a reduction in inflammation is seen and consequently this research concurs with other studies suggesting that APPs should not be used as the sole physiological measure of health and welfare at the abattoir. Levels of cortisol measured from hair samples can reflect several months of cortisol secretion, and the inclusion of hair cortisol levels as a welfare measure at slaughter is useful in this context. The current findings should be validated in a larger-scale trial involving commercially-farmed pigs, and additional physiological measures evaluated, before the inclusion of these measures into routine MI processes can be recommended.
